# P293: Infectious risk assessment in the use of digestive endoscopy to hospital in Abidjan

**DOI:** 10.1186/2047-2994-2-S1-P293

**Published:** 2013-06-20

**Authors:** C Assi, MF Adeoti

**Affiliations:** 1 Department of Gastroenterology, CHU of Cocody, Abidjan, Côte d'Ivoire; 2 Central Laboratory , CHU Yopougon, Côte d'Ivoire

## Introduction

Gastroscopy can be a source of contamination and can lead to nosocomial bacterial infections. The majority of these infections are caused by inadequately cleaned and disinfected endoscopes.

## Objectives

To evaluate bacteriological samples from the practices of cleaning and disinfection in 11 endoscopy centers in Abidjan.

## Methods

A prospective, multicenter, cross-sectional, six-month study in 11 of the 30 endoscopy centers in Abidjan (equipment room gastroscopy, protective equipment and qualification of personnel, conditions of cleaning, decontamination, disinfection, rinsing, drying and storage gastroscopes, bacterial samples).

## Results

It was observed: i) 100% of decontamination and disinfection of endoscopes were carried out by unskilled personnel, ii) 82% of the centers used 2% glutaraldehyde and 73% quaternary ammonium compound with a minimum time of 30 minutes for decontamination and disinfection iii) One center (9%) changed the rinse water after the passage of each patient, iv) 91% of the centers visited were within the concentration recommended by the manufacturers of the products decontamination and disinfection, v) 100% of the centers performed gastroscope and other equipment sampling with positivity in 10 of 11 centers, primarily Bacillus in rinsing tanks.

## Conclusion

Disinfection of gastroscopes is poor in endoscopy centers in Abidjan because of the presence of germs found in almost all the centers evaluated. Reduced risk of infection requires establishing standards, staff training and regular quality control.

## Competing interests

None declared

